# The dark side of Tregs during aging

**DOI:** 10.3389/fimmu.2022.940705

**Published:** 2022-08-09

**Authors:** Martina Palatella, Stephane M. Guillaume, Michelle A. Linterman, Jochen Huehn

**Affiliations:** ^1^ Department Experimental Immunology, Helmholtz Centre for Infection Research, Braunschweig, Germany; ^2^ Immunology Program, The Babraham Institute, Cambridge, United Kingdom; ^3^ Cluster of Excellence RESIST (EXC 2155), Hannover Medical School, Hannover, Germany

**Keywords:** regulatory T cells, aging, pathogen-specific immune response, vaccination, germinal center reaction

## Abstract

In the last century, we have seen a dramatic rise in the number of older persons globally, a trend known as the grey (or silver) tsunami. People live markedly longer than their predecessors worldwide, due to remarkable changes in their lifestyle and in progresses made by modern medicine. However, the older we become, the more susceptible we are to a series of age-related pathologies, including infections, cancers, autoimmune diseases, and multi-morbidities. Therefore, a key challenge for our modern societies is how to cope with this fragile portion of the population, so that everybody could have the opportunity to live a long and healthy life. From a holistic point of view, aging results from the progressive decline of various systems. Among them, the distinctive age-dependent changes in the immune system contribute to the enhanced frailty of the elderly. One of these affects a population of lymphocytes, known as regulatory T cells (Tregs), as accumulating evidence suggest that there is a significant increase in the frequency of these cells in secondary lymphoid organs (SLOs) of aged animals. Although there are still discrepancies in the literature about modifications to their functional properties during aging, mounting evidence suggests a detrimental role for Tregs in the elderly in the context of bacterial and viral infections by suppressing immune responses against non-self-antigens. Interestingly, Tregs seem to also contribute to the reduced effectiveness of immunizations against many pathogens by limiting the production of vaccine-induced protective antibodies. In this review, we will analyze the current state of understandings about the role of Tregs in acute and chronic infections as well as in vaccination response in both humans and mice. Lastly, we provide an overview of current strategies for Treg modulation with potential future applications to improve the effectiveness of vaccines in older individuals.

## 1 Introduction

Over the past century, we have seen an unprecedented increase in the human lifespan. The World Health Organization estimated that the number of individuals aged 60 or over in 2020 was 1 billion, a number anticipated to rise to 1.4 billion by 2030 and 2.1 billion by 2050. Furthermore, the number of people over the age of 80 is expected to triple between 2020 and 2050, reaching an estimated 426 million (1). This population aging or grey (silver) tsunami (2) is largely due to significant lifestyle improvements and to the great progress made in the fields of modern medicine and experimental research.

The major challenge associated with population ageing is that this increase in the duration of lifespan is associated with more people experiencing age-related diseases (3), which can be attributed in part to the physiological functional decline of several organs, tissues, and bodily systems. Among these, the immune system is affected and, as it permeates almost all tissues, immune ageing can influence nearly every organ in the body. The age-related immunological changes can be summed up in two major events. One is a chronic, low-grade, systemic inflammation, often referred to as inflammaging, which occurs in the absence of pathogens across various tissues. The other encompasses the progressive decline and dysfunction of the immune systems’ competence with age, known as immunosenescence, affecting both the innate and adaptive arms of immunity (4). Inflammaging and immunosenescence are two sides of the same coin, as they mutually interact with each other and co-evolve in response to external stimuli that shape the immunobiography of each individual (5). Consequently, the concept of biological age has recently acquired great importance in the aging field to highlight that the real age is not the chronological, but the biological one (6). Together, these two immune aging phenomena are thought to be risk factors leading to greater susceptibility to infections, autoimmune diseases, malignant tumors, and multimorbidity, as well as reduced response to vaccination, all of which are typically observed among the elderly population (7). This phenomenon has become particularly evident during the coronavirus disease (COVID-19) pandemic, where aged individuals are disproportionally more at risk of severe disease and death after severe acute respiratory syndrome coronavirus 2 (SARS-CoV-2) infection compared to younger cohorts (8, 9). Another important aspect to consider is that this demographic revolution not only represents a major health concern, but also poses a significant challenge for our health care system to provide high-quality care for this fragile portion of the population (10). Consequently, it is vitally important to unravel the impact that aging has on our immune system, to develop preventive and therapeutic therapies which can better protect the elderly.

Current knowledge of the molecular mechanisms behind these age-related immune system changes is still limited. However, a growing body of literature support the hypothesis that age-related gut dysbiosis could contribute to immune aging (11, 12). Considerable perturbations in the microbiota composition, including a shift towards bacteria with more pro-inflammatory properties, as well as diminished intestinal epithelial barrier integrity have been identified in old fruitflies (13), mice and humans (14). Fransen *et al.* demonstrated that fecal microbiota transplantation (FMT) from young or old mice to germ-free (GF) mice results in significant broad changes in the resulting immunophenotype, showing increased frequencies of T-helper (T_H_) 1, T_H_2 and Tregs cells in the spleen, and T_H_1 in the Peyer’s patches of GF mice which received the ‘old’ microbiota (15). They also noted enhanced Toll-like receptor 2 activation, suggesting an increased translocation of bacterial products from the gut lumen to the circulation. Consistent with this, Thevaranjan *et al.* observed that enhanced leakage of the gut bacteria to the circulation subsequently results in systemic inflammation, as indicated by higher serum levels of tumor necrosis factor (TNF)-α and interleukin (IL)-6 (16), thus contributing to the systemic inflammatory state seen in the elderly. Interestingly, it has also been reported that in older people there is a defect in the formation of circulating T follicular helper (T_FH_) cells upon vaccination, and this was correlated with an enhanced inflammatory gene signature rather than a contraction of the T cell receptor (TCR) repertoire (17, 18). Of note, Stebegg *et al.* showed that heterochronic FMT from young into old mice and vice versa, boosts germinal center (GC) reaction in Peyer’s patches irrespective of age directionality (19). Instead, this boost does not occur upon cross-strain FMT from BALB/c to C57BL/6 mice of the same age (19). Overall, findings from this study suggest that the GC reaction in Peyer’s patches responds specifically to heterochronic FMT and is not uniquely driven by young microbiota transplanted in an aged host. Among the many age-related immunological changes, several studies have reported an increase in the frequency of Tregs in secondary lymphoid organs (SLOs) (20–24). These CD4^+^CD25^+^ lymphocytes are characterized by a high expression of forkhead/winged-helix family transcription factor (Foxp3) and, in humans, by low levels of the alpha chain of IL-7 receptor (CD127). Tregs exert immunosuppressive functions which are fundamental in preventing deleterious effects caused by either hyperstimulation or autoreactivity of the immune system (25). As such, it has been proposed that age-related changes in Tregs may be a contributing factor for the increased vulnerability to different diseases observed in older people. Besides the most common CD4^+^Foxp3^+^ Tregs there are also CD8^+^Foxp3^+^ Tregs, whose properties have been recently comprehensively summarized by Liston and Aloulou (26), and populations of Tregs that stably reside within tissues, known as tissue-resident Tregs. Here, we will focus on CD4^+^Foxp3^+^ Tregs and provide an update on previous reviews (27–30) about the current understanding of the role SLO-derived and tissue-resident Tregs play in acute and chronic infections, as well as in vaccine-induced immune responses, with a focus on aged mice and older humans. In addition, we address current approaches being explored to modulate Treg activity, with potential future applications in vaccines tailored towards elderly individuals.

## 2 Tregs in aging: Quantitative changes

Although the existence of lymphocytes with suppressive activity was proposed in the early 1970s (31), the first phenotypic marker identified for these cells, IL-2 receptor alpha chain or CD25, was discovered 25 years later in mice (32). After that, several groups simultaneously reported the presence of a CD4^+^CD25^+^ T cell population with immunosuppressive activity in humans (33–37). In the early 2000s, the transcription factor Foxp3 was identified as lineage-specifying factor determining the unique properties of these immunosuppressive cells, and from that moment on, it became the predominant marker for Treg identification (38–40). The function of Tregs in immune suppression has been described in detail in previous reviews (41–43). In brief, Tregs suppress immune responses through four dominant pathways: *I)* the release of inhibitory cytokines, including transforming growth factor (TGF)-β, IL-10 and IL-35, to reduce effector T (Teff) cell activity (44–47), *II)* direct cytolysis of Teff cells (48, 49), *III)* metabolic disruption by acting as a sink for T cell growth-stimulating cytokines, such as IL-2 (50, 51), and *IV)* targeting and inhibiting dendritic cells (DCs) e.g. through expression of membrane-bound receptors like cytotoxic T-lymphocyte associated protein 4 (CTLA-4) and lymphocyte-activation gene 3 (LAG3) (52–54).

**Figure 1:**
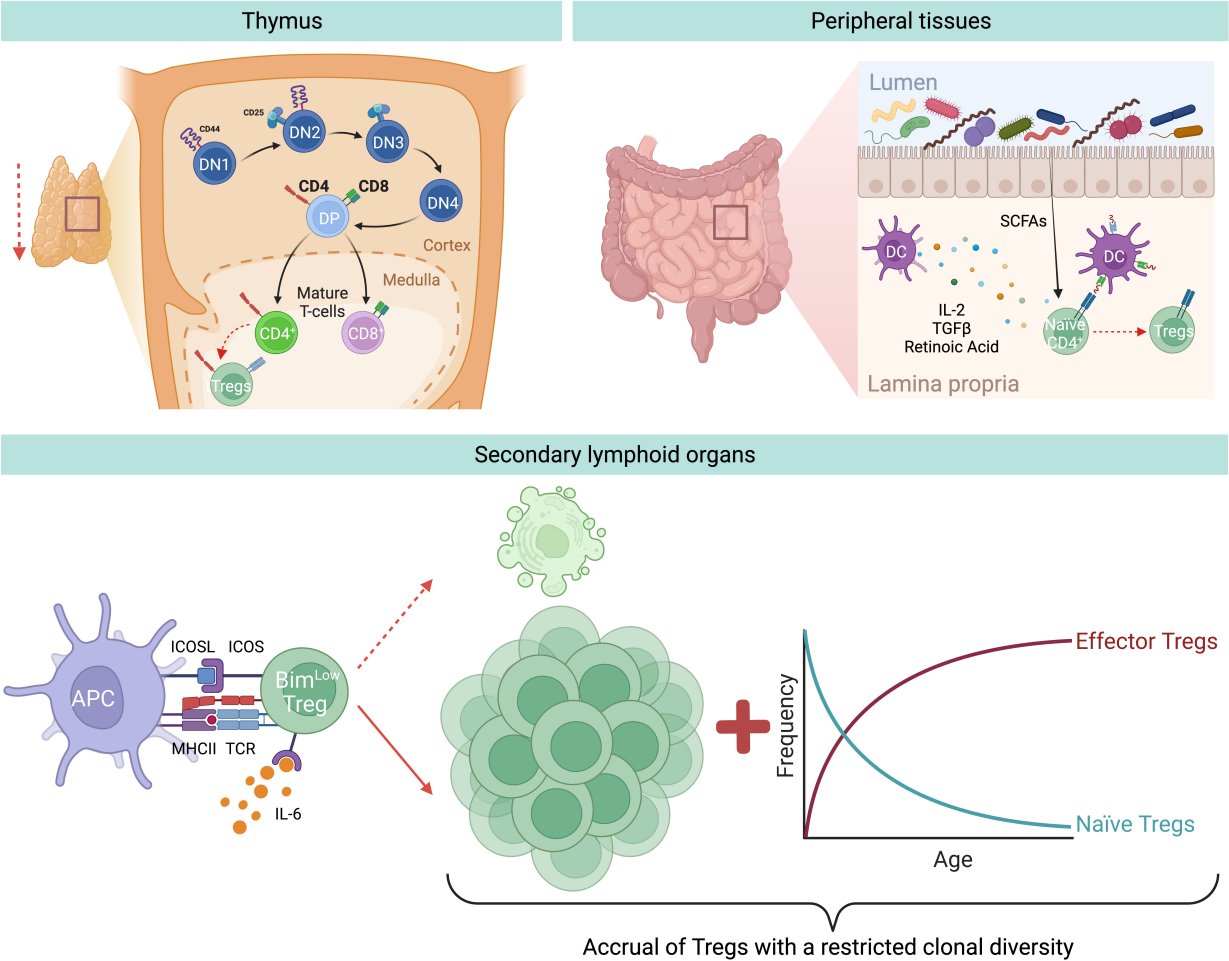
Aging-related changes of Treg generation and homeostasis. During aging there is a decreased generation of tTregs and pTregs. Thymic involution and reduced input of progenitor cells from the bone marrow result in a reduced output of recent thymic emigrants, among which are also less tTregs. In addition, in the peripheral tissues, for instance in the gut, there is a reduced induction of pTregs from naïve CD4^+^ T cells, likely due to age-associated changes in bacterial-derived metabolites, e.g. short chain fatty acids (SCFAs), or DC-derived Treg-inducing molecules, e.g. TGF-β or retinoic acid. Nevertheless, during aging there is an accumulation of Tregs in SLOs. This is mainly attritubed to the enhanced ability of old Tregs to escape apoptosis by selectively downregulating the expression of the pro-apoptotic protein Bim *via* the IL-6-ICOS-Bim axis. In addition, owing to the decreased production of naïve Tregs from the thymus, there is a resulting increase of effector Tregs in the elderly.

Tregs can originate from either the thymus or are generated at peripheral sites (**Figure 1**). Those produced in the thymus are termed thymic Tregs (tTregs). Within this primary lymphoid organ, the cellular branch of the immune system is educated through thymic negative selection, where some self-reactive CD4^+^ T cells differentiate and become tTregs. However, during aging, the thymus undergoes a gradual reduction in size and function, together with changes in its architecture both in mice and humans. This phenomenon is referred to as thymic involution and is characterized by the progressive deterioration and disappearance of the thymic cortex and medulla, and replacement with adipose tissue (55). Hence, only traces of functional thymic tissues are typically found in people over 70 years old (55). The exact causes behind this thymic involution, however, are not fully understood. Among the possible factors, there might be age-related changes in the levels of thymo-stimulatory growth hormones, including decreased levels of growth hormone (GH) and insulin-like growth factor (IGF)-1, in addition to changes in steroid hormones, thymo-suppressive and inflammatory cytokines (e.g. increasing levels of IL-6), and oxidative stress-induced damage (56). Furthermore, a reduced number of bone marrow progenitors reaches the thymus, and the thymic epithelial cell (TEC) number decreases with age. Interestingly, adipocytes are not only responsible for anatomical changes within the thymus, but they actively contribute to thymic involution by producing negative factors for thymic maintenance (e.g. IL-6, sex hormones, and steroids), thereby transmitting suppressive signals to TECs, reducing thymopoiesis and cellularity (57). Despite the decline in thymic cellularity, no blockage of thymocyte differentiation is observed, since thymic function is maintained in proportion to the reduced size both in mice and humans (58, 59). The outcome of thymic T cell reduction is a correlative decrease in tTreg production with age (60).

Another important Treg population originates at peripheral sites, referred to as peripheral Tregs (pTregs), which are important for promoting tolerance towards non-pathogenic foreign antigens (e.g. food and commensal antigens) (**Figure 1**). Similarly to tTregs, pTreg differentiation appears to diminish with age, demonstrated by several studies which reported that naïve conventional T cells (Tconv) from aged mice differentiate less into pTreg *in vivo* and *in vitro* compared to Tconv from young animals (61–63). Takano *et al.* observed a similar reduction in the *de novo* induction of antigen-specific Tregs in the intestines of aged mice compared to their young counterparts, when using a Treg induction model where *Rag2* ^-/-^ mice bearing ovalbumin-specific TCR-transgenic T cells were fed a diet containing ovalbumin (OVA) (64). Moreover, the group analyzed DCs in the mesenteric lymph nodes (MLN) and found a significant downregulation in retinaldehyde dehydrogenase 2 (RALDH2) gene expression in MLN DCs from aged mice, as well as a reduced frequency of CD11b^–^CD103^+^PD-L1^high^ DCs, a subset characterized by the highest expression of this enzyme (64). RALDH2 is involved in the synthesis of retinoic acid which allows MLN DCs to foster the induction of Tregs, together with TGF-β (65). Thus, the authors hypothesize that the reduced expression of this enzyme may be a contributing factor to the reduced pTreg generation observed during aging. Interestingly, DNA methylation analysis revealed greater methylation of the CpG motifs in the *RALDH2* promoter region in MLN DCs of aged mice, suggesting that this down-regulation is due to age-dependent epigenetic changes (64). Despite reduced tTreg and pTreg generation, studies conducted on multiple strains of mice showed increased Treg prevalence in SLOs (spleen and lymph nodes) of aged mice compared to young animals (21, 66). This accumulation seems to progress with age, as middle-aged mice show intermediate levels of Tregs when compared to young and old mice (62, 67). In humans the situation is less clear since, owing to the limited access of tissues, most of our knowledge comes from analyses of human blood, where only a modest increase in the frequency of circulating Tregs was observed. In particular, an increased frequency of activated Tregs (FOXP3^hi^CD45RA^-^) and a low frequency of resting Treg population (FOXP3^low^CD45RA^+^) were detected in the blood of old respect to young humans (66, 68, 69), most likely because these cells are sequestered in tissues. The only study evaluating the frequency of Tregs in human tissues showed a higher frequency of Tregs in the skin of old people compared to their younger counterparts (70), thus suggesting that Tregs accumulation also occurs in elderly humans. Of relevance, the increased percentage of Tregs in the skin biopsies was observed both pre- and post-antigenic stimulation, thereby pointing out that not only Treg numbers are higher under homeostatic conditions in older adults, but also they can further expand upon antigenic stimulation.

These findings led to the hypothesis that during aging Tregs accumulate in SLOs, thus hypothetically explaining both the increased susceptibility to infections and the reduced vaccine response in the elderly (71). Given that this age-driven Treg accumulation is not due to increased Treg generation, this phenomenon could alternatively be explained by either enhanced cell proliferation or increased survival. The former hypothesis was falsified, as Tregs from old mice show a reduction in proliferation compared to Tregs from young mice (62, 72). Instead, the latter hypothesis was confirmed, as Chougnet *et al.* observed that aged Tregs survive longer than their younger counterparts (62). This enhanced survival is due to the selective loss of expression of the pro-apoptotic protein Bim in Tregs with age, thus making old Tregs more apoptosis-resistant compared to those from young individuals (62) (**Figure 1**).

Another possible explanation for the accumulation of Tregs in older individuals could be the age-related accrual of mature Tregs at the expense of naïve CD4^+^ T cells. In support of this, Thiault *et al.* discovered that activated antigen-experienced Tregs with effector/memory phenotype return to the thymus from the periphery and suppress the generation of new tTregs (73), most likely by competing for the IL-2 required for their development (50, 74). Consequently, the aged Treg pool is skewed towards effector/memory rather than naïve phenotype (**Figure 1**), resulting in reduced clonal diversity, which further translates into suppression only of certain T cells, while leaving the rest unaffected. Overall, this could favor some pro-inflammatory cells to remain active in aged hosts (28). Additionally, age-related changes to the endocrine profile may contribute to the accumulation of Tregs during aging. Indeed, it has been demonstrated that glucocorticoids, which are increased in the elderly population, can enlarge this regulatory population in the periphery (75).

Age-related changes in the cytokine production may also contribute to Treg accumulation. It is well-known that circulating IL-2 levels decrease with age and in line with this, Raynor *et al.* observed that most of the accumulating Tregs in aged mice express low levels of CD25 and higher levels of CD122 (the beta chain of the receptor for IL-2 and IL-15), suggesting that they are less dependent on IL-2 for their survival and rely more on IL-15 (67). Gene expression profiling of these CD25^lo^ Tregs in aged mice revealed that they have an effector phenotype characterized by high levels of inducible T cell co-stimulator (ICOS) and CD69 (72). Of note, *in vivo* antibody blockade of ICOS ligand (ICOSL) led to a loss of effector Tregs (eTreg), and this loss was rescued in Bim-deficient mice (72), revealing that ICOS is critical for eTregs maintenance, likely by inhibiting Bim-mediated cell death (**Figure 1**). In addition, *ex vivo* experiments revealed that IL-6 exerts an additive effect on ICOS expression in combination with TCR stimulation, while IL-6-deficient aged mice show a significant loss of eTreg and reduced ICOS expression. Hence, an increased level of circulating IL-6, as seen in older individuals (76), is critical for eTreg accrual, likely by enhancing TCR-driven ICOS expression (72). Altogether, these results highlight a novel role for the pro-inflammatory cytokine IL-6 during aging: on one hand it promotes inflammaging; on the other hand, it counterbalances this inflammation by elevating Treg numbers. Although it is well documented that the number of Tregs in SLOs increases with age, the exact molecular mechanisms behind the regulation of aged Tregs *via* this IL-6-ICOS-Bim axis remain to be understood.

## 3 Tregs in aging: Qualitative changes

With regard to Treg immunosuppressive activity, there is still an open debate regarding whether these cells have increased or decreased functionality during aging. In line with the hypothesis of Tregs having a reduced suppressive capacity, the adoptive transfer of Tregs from aged to young recipient mice resulted in a greater incidence of delayed-type hypersensitivity responses compared to the infusion of young Tregs (21). Analogously, Guo et al. found that in comparison with young Tregs, aged ones were less efficient in proliferating and suppressing Tconv function both *in vitro* and in an inflammatory bowel disease model (77). They also showed that Tregs preferentially senesce over Tconv through DCAF1/GSTP1/ROS-dependent mechanisms (77). Consequently, there is a functional imbalance between Tregs and Tconv during aging, which favors Tconv activation and inflammation. The group proposed the GSTP1/ROS axis as a molecular target for reinvigorating Treg function and restraining immunological aging (77). Tsaknaridis *et al.* published similar findings in humans, as they observed a significant loss of Treg suppressive activity in some donors older than 50 years, which were found to have almost ten-fold less suppressive activity than Tregs from younger donors (78).

However, there is also evidence from other studies that the suppressive activity of Tregs is not contingent on age, but instead is retained at nearly the same level throughout the lifespan. These studies showed that Tregs from young and aged mice (20) as well as in humans (69, 79) have the same suppressive capacity since they can suppress CD4^+^ T cell proliferation to the same extent. Similarly, Williams-Bey *et al.* reported that CD8^+^ T cell proliferation and interferon (IFN)-γ production are suppressed to the same extent by Tregs derived from aged compared to young mice (80).

On the other hand, Tregs from old mice have also been described as being better suppressors than those from young adult animals due to higher IL-10 production (22). Garg *et al.* have investigated the molecular mechanisms underlying this enhanced functionality and discovered that it correlates with an increased Foxp3 expression level driven by the hypomethylation of CpG sites within the *Foxp3* locus (22). Of note, aged Tregs can also limit the availability of extracellular cysteine, thus leading to an imbalanced redox potential that is unfavourable for T cell activation and proliferation (22).

The most likely reason behind these inconsistencies and conflicting data is that CD25 does not accurately recapitulate Foxp3 expression and Treg activity in aged mice, so it may act as a confounding factor in the interpretation of suppression assays (20). To circumvent this caveat, Lages et al. used Foxp3^GFP^ reporter mice and observed that Tregs from old mice have a greater *in vitro* suppressive activity on a per cell basis than their younger counterparts (66). Another major caveat regarding the use and interpretation of *in vitro* suppression assays is that they may not be truly representative of *in vivo* Treg-mediated suppression mechanisms (81). Results obtained through the use of anti-CD25 antibodies do not allow researchers to discriminate between the suppressive activity of CD4^+^ and CD8^+^ Tregs, which have also been reported to increase with age (82). Results can also be deeply influenced by the responder cells, which may change depending on the cell’s age and type, as well as the stimuli they receive, independently of Treg functions (83). Moreover, the *in vivo* model used might affect Treg suppression, phenotype and homing, depending on the unique local inflammatory environment (84, 85). In human studies, suppression assays are often performed with CD25^+^ Tregs isolated from peripheral blood. Within this CD25^+^ population, there may not be only Tregs, but also recently activated Tconv, while the majority of aged CD25^lo^ Tregs might not be included. Additionally, circulating Tregs might not be representative of the tissue-resident ones, as differential expression levels of CTLA-4 have also been reported (66). Another important point to keep in mind is that whereas in mice Foxp3 expression is limited to Tregs, in humans this is not true, since recently activated T cells without suppressive capacity can also express low level of FOXP3 (29). Therefore, Miyara *et al.* proposed a new classification of human Tregs into three populations, based on the expression of FOXP3 and CD45RA: (I) FOXP3^low^CD45RA^+^ as resting Tregs, (II) FOXP3^high^CD45RA^-^ as activated/effector Tregs and (III) FOXP3^low^CD45RA^-^ as non-suppressive, cytokine-producing non-Tregs (86). Thus, it is important to combine FOXP3 expression with other markers in order to identify human Tregs. For cell isolation, CD25 can be used in combination with CD127 (87) or CD45RA (86) to achieve a better separation of naive Tregs, effector Tregs and CD25^+^ Tconv.

Taken together, these conflicting data do not completely explain the simultaneously increased risk of autoimmunity, cancer, and infections observed in the elderly. Therefore, how the intrinsic functions of Tregs change during aging and what the impact of those changes may be remain questions yet to be elucidated (88).

## 4 Tissue-resident Tregs: Qualitative and quantitative changes

During the recent past, much attention has been paid to tissue-resident Tregs, a subset of Tregs that differ from their SLO-derived counterparts due to their residence within non-lymphoid tissues, reduced TCR repertoire diversity, and completely distinct transcriptomes and functional properties (89). Tissue-resident Tregs can be found in several tissues, for instance in the visceral adipose tissue (VAT), skeletal muscles, central nervous system, colon and small intestine lamina propria, where they are involved in the maintenance of tissue integrity and functionality. Overall, tissue-resident Tregs accumulate in tissues from birth till adulthood, but their number suddenly decreases in aged individuals (89). Although the reasons for this decline are not elucidated yet, the consequences on tissue homeostasis and functions are severe. Therefore, future studies on tissue-resident Tregs and their relative age-related modifications are urgently needed.

### 4.1 VAT-Tregs

In a seminal study, Feuerer et al. (90) identified a unique population of fat-resident Tregs in mouse and human. Surprisingly, unlike SLO-derived Tregs, which represent 10-15% of the CD4^+^ T cell compartment, more than 50% of CD4^+^ T cells found in the epididymal fat pads express Foxp3. These Tregs have suppressive activity equal to that of SLO-derived Tregs, but display a different transcriptional profile (90). VAT-Tregs are thought to originate from the thymus, since they express high levels of Helios and neuropilin-1 (Nrp-1), which are markers for tTregs (91). In line with this, adoptively transfered Tconv do not give rise to VAT-Tregs (92). Interestingly, the master transcription factor of adipocytes, peroxisome proliferator-activated receptor gamma (PPAR-γ), is also a major orchestrator of the unique properties of VAT-Tregs, including cell accumulation, phenotype, and functions (93, 94). To evaluate the importance of *Pparg* in VAT-Tregs, Cipolletta *et al.* generated a conditional mouse line lacking *Pparg* expression exclusively in Tregs (93). Notably, in these mice they observed a decrease in VAT-Treg number and a loss of their function, while Tregs from other tissues were not affected (93). Moreover, depletion of VAT-Tregs in lean mice resulted in VAT inflammation and metabolic alterations, suggesting that VAT-Tregs play an important role in the adipose tissue metabolism (93). Likewise, administration of PPAR-γ agonists to mice fed with a high-fat diet led to VAT-Treg expansion and helped ameliorating local inflammation and metabolic health (93). Besides *Pparg*, VAT-Tregs also express high levels of *Il1rl1* encoding for the IL-33 receptor, also known as ST2 (93), suggesting that IL-33 may play an important role for VAT-Tregs. Indeed, it has been demonstrated that IL-33 strongly promotes the accumulation of VAT-Tregs (91, 95).

Treg accumulation in the VAT seems to occur early in life (prior to 3-4 weeks of age), since thymectomy at this age does not affect the number of VAT-Tregs (91). Interestingly, monitoring of VAT-Tregs isolated from epididymal fat depots of C57BL/6 mice during aging revealed an evident increase of this population at 14 weeks of age, reaching a peak at 25 weeks. Instead, at older ages (40 weeks), VAT-Tregs drop rapidly, accompanied by a decline in insulin sensitivity (96). Of note, microarray-based gene-expression profiling of VAT- and lymph node-derived Tregs from C57BL/6 male mice of 5, 14, 25 and 40 weeks of age revealed that, analogously to the dynamics of VAT-Treg numbers during aging, the number of differentially expressed genes rise from 5 to 14 weeks, and peak at 25 weeks, while drastically shrank at 40 weeks of age (96). This evolution of the unique VAT-Tregs’ transcriptome likely reflects a progressive adaptation of this population to the adipose tissue microenvironment throughout life. In contrast, this does not occur in Tregs isolated from lymph nodes, which show very few differences in their transcriptome from 5- and 25-weeks-old mice. Overall, SLO-derived Tregs preserve their transcriptome during aging, while VAT-Tregs show a progressive transcriptomic evolution. Regarding the rapid decrease in the number of differentially expressed genes in VAT-Tregs starting at very old ages, it remains to be elucidated whether this corresponds also to a loss of function or dysfunction of VAT-Tregs in old animals.

Age- and obesity-associated insulin resistance are two physiologically distinct forms of adult-onset diabetes. While it is well-known that macrophage-driven inflammation drives obesity-associated insulin resistance (97–101), the underlying mechanisms for the age-associated insulin resistance are largely unknown. Given that insulin resistance is prevalent in the elderly population, the VAT-Tregs frequency reduction observed at old ages might contribute to increase this susceptibility. In fact, in obese individuals, the number of Tregs in the circulation as well as in VAT is substantially reduced (102), and this is closely associated with an increase in inflammatory mediators and a decrease in insulin sensitivity in the VAT (103). In contrast, supplementation with Tregs reduces VAT inflammation and improves metabolic parameters in obese mice (103).

Most of the data reported here stem from experiments performed on C57BL/6 male mice. Strikingly, in contrast to these findings, one study reported a greater accumulation of VAT-Tregs in old mice, suggesting a completely different scenario (104). Of note, they also demonstrated that depletion of VAT-Tregs in old mice protects them against age-associated insulin resistance, yet they remain susceptible to obesity-associated insulin resistance and metabolic disease (104). In line with this, selective depletion of VAT-Tregs *via* anti-ST2 antibody treatment increases adipose tissue insulin sensitivity in aged mice (104). This theory according to which VAT-Tregs keep on accumulating even in the aged adipose tissue, is in concordance with the ‘adipose tissue expandability’ hypothesis, which proposes that in the context of positive energy balance, it is not the absolute fat mass that determines the appearance of metabolic complications, but instead the inability of white adipose tissue to further expand and appropriately accommodate energy surplus (105). In aged mice, the excessive accumulation of VAT-Tregs leads to suppression of the healthy inflammation required for remodeling of adipose tissue. This further results in a storage space problem leading to ectopic deposition of fat in visceral organs, with subsequent free fatty acid induced lipotoxicity and age-associated insulin resistance (106).

Possible explanations behind these discrepancies in age-related quantitative changes in VAT-Tregs might depend on different murine colonies, husbandry practices, and dietary composition (104). Lastly, Laparra *et al.* have identified discrepancy on VAT-Tregs among mouse models, cynomolgus macaques and humans (107). In particular, the proportion of VAT-Tregs is low in all these species, except for C57BL/6 mice, which show male-specific and aging-related increase of Tregs (107). Therefore, these differences should be taken into account when translating these results to different species.

### 4.2 Skeletal muscle Tregs

The presence of Tregs within skeletal muscles was reported for the first time in 2013 (108). Under homeostatic conditions, skeletal muscle Tregs represent a minority of the CD4^+^ T cell compartment (about 10%), but upon injury, they rapidly expand to support tissue repair (108, 109). The main driver of Treg accumulation within injured skeletal muscles is the alarmin IL-33, which is secreted by cells resembling fibro/adipogenic progenitor cells in response to tissue injury (109). IL-33 binds to ST2, which is highly expressed by muscle Tregs, and in response they promote tissue repair by releasing the epidermal growth factor receptor (EGFR) ligand amphiregulin (AREG), that directly act on satellite cells (108, 109). The origin of skeletal muscle Tregs has not been elucidated yet. Treatment with the sphingosine-1-phosphate receptor 1 agonist FTY720, before inducing muscle injury, reduced the absolute number of accumulated Tregs, but not their proportion among CD4^+^ T cells in the muscle (109), suggesting accumulation of muscle Tregs in response to injury may be dependent on the recruitment from the circulating T cell pool. However, this does not exclude the possibility of a local expansion of Tregs that are already residing in the muscle.

During aging, skeletal muscles undergo a progressive decline, affecting both their function and regeneration capacity. Indeed, old people are prone towards muscle wasting and musculoskeletal injury. This can be partially explained by the age-related reduction of satellite cells, a pool of multipotent precursor cells. However, age-related changes affecting skeletal muscle Tregs might also play a role. In uninjured skeletal muscle of young and old C57BL/6 mice, muscle Tregs represent a small proportion of the CD4^+^ T cell compartment in both mice (109). However, upon injury, skeletal muscle Tregs rapidly expand to constitute up to 40-50% of CD4^+^ T cells in young mice, while in old mice such an increase does not occur (108). This reduced skeletal muscle Tregs accrual is attributed to age-related defects in their recruitment from SLOs, proliferation and retention within the muscles (109). Moreover, in old mice, IL-33 signalling is significantly blunted due to reduced IL-33 expression and reduced number of IL-33 secreting fibro/adipogenic progenitor-like cells (109). Of note, IL-33 injection not only rescues Treg function, but also improved muscle regeneration, by triggering satellite cell expansion and a shift of the muscle transcriptome towards a muscle repair signature (109).

Little is known about skeletal muscle Tregs in humans however, if confirmed, this decreased Treg accumulation in old people may contribute to sarcopenia (110). Thereby, further in-depth studies will be fundamental to unravel the molecular mechanisms behind this detrimental condition, which is highly prevalent among the elderly.

### 4.3 Colonic Tregs

The gastrointestinal tract is the site of food nutrients absorption as well as a place where a mutual symbiotic relationship is established between the immune system and the microbiota. Therefore, the intestinal immune system has the complex task of promoting tolerance toward food antigens (oral tolerance) (111, 112) and commensal bacteria, and at the same time it provides barrier immunity against pathogenic bacteria. Tregs are crucial mediators of intestinal tolerance and tissue homeostasis. Specifically, in the colonic lamina propria, two distinct populations of colonic Tregs can be identified based on transcription factor expression: GATA3^+^Helios^+^ and RORγt^+^ Tregs (113). Colonic Tregs have a mixed origin, since GATA3^+^Helios^+^ Tregs largely constitute of tTregs, while RORγt^+^ Tregs mainly consist of pTregs. In general, colonic Tregs recognize gut microbiota-derived antigens (114, 115), in particular colonic RORγt^+^ Tregs are primarly induced by microbial antigens (116–118), supported by short-chain fatty acids (119–121), TGF-β (122, 123), and the vitamin A derivative retinoic acid (116). It has been hypothesized that this subpopulation of colonic Tregs controls local inflammatory responses, however it is still not clear whether they are focused only on type 2 reactions (116) or if they have a more general purview (117, 118).

Upon broad-spectrum antibiotic treatment, colonic Treg numbers decrease (124), but do not completely disappear. Indeed, GATA3^+^Helios^lo/-^ colonic Treg induction and acccumulation is less depedent on the gut microbiota composition, while it relies more on IL-33, as they express ST2 (125). These colonic GATA3^+^Helios^+^ Tregs participates in local repair processes (126), given their induction by the alarmin IL-33 and their consequent production of the tissue-repair factor AREG (125).

Interestingly, there is a time-controlled development of the two colonic Treg subsets (127). Early in life, GATA3^+^Helios^+^ Tregs are the dominant subset, since there are only few bacterial species in the gut, most of them are Lactobacillus commensals from breast milk, and they are maily controlled by maternal IgA. Instead, around weaning, three weeks after birth in mice, the nutritional input changes considerably from a milk-only diet to one that including solid food. Consequently, there is a rapid expansion of the gut microbiota both in numbers and richness of bacterial species.

In this moment in life, a window of opportunity has been identified, during which the host mounts the first vigours immune response to colonizing intestinal microbiota, the so-called weaning reaction (128). If this early-life window of opportunity is missed, due to lack of exposure to microbiota or exposure to a dysbiotic microbiota, then a pathological imprinting of the immune system can occur, resulting in increased susceptibility to inflammatory pathologies (129) or altered response to vaccination later in life (130). During the weaning reaction, RORγt^+^ Tregs become dominant, since maintenance of immunological tolerance to commensals becomes paramount. Interestingly, Al Nabhani *et al.* showed that these microbiota-induced RORγt^+^ Tregs must be generated during the weaning reaction in order to prevent pathological imprinting of the immune system (128), e.g. to prevent spontaneous Th2-mediated allergic responses to dietary antigens (131) and protect from dextran sodium sulfate (DSS)-mediated severe colitis in mice (132). Regarding humans, the knowledge is still very limited. GATA3^+^ Tregs have been observed only in human blood so far (133), while RORγt^+^ Tregs were identified in human colonic lamina propria from both healthy controls and patients with Crohn disease (117). Nonetheless, it remains to be clarified to what extent these phenotypes parallel the murine subsets mentioned above.

## 5 Acute and chronic infections in aging

Surviving a given infection requires the generation of a controlled immune response, which on one side should be strong enough to eradicate the invading pathogen, but on the other side, it should not be too exaggerated to avoid collateral tissue damage. The role of Tregs during infections is quite controversial. Some studies suggest that the suppressive nature of Tregs hinders the immune response to infection, therefore they are detrimental to the host (134–138), while other studies have shown that Tregs are crucial for the successful eradication of the invading pathogens (139–145). Overall, Tregs play a dual role during infections: they are beneficial for the host since they keep pathogen-specific immune responses under control, but in this way they also facilitate chronic pathogen persistence by reducing effector immunity and clearance of infection (146). During acute infection, the beneficial role of Tregs seems to predominate. For instance, in genital herpes and respiratory syncytial virus infections, Tregs control excessive chemokine accumulation (e.g., IFN-α and -γ) in the draining lymph nodes, thus regulating the entry of Teff cells, DCs and natural killer cells at the infection site, thereby indirectly controlling the growth and spread of infection (141, 147). In addition, Tregs can suppress infected cells’ proliferation so that infection cannot be established (148, 149), and are also crucial in promoting memory formation by increasing the time window of antigen availability (150). In contrast, in a mouse model of bacterial infection with *Salmonella enterica* serotype Typhimurium, Tregs plays a negative role by suppressing anti-Salmonella responses early after infection and thus allowing invasion of the pathogen (136). Anyway, the complete eradication of the infection can be achieved at later time points in concomitance with a decrease in the Tregs’ suppressive capacity (136). Otherwise the pathogen may achieve a carrier state of persistent asymptomatic infection, acting as a shedded reservoir for subsequent re-infections (151). This is also valid for *Helicobacter pylori* infection, whose carrier state can last life-long, and its ability to induce Tregs, which may appear beneficial initially, could later on lead to chronic inflammation and tumor induction (152, 153).

During chronic infections, the functions of Tregs are contradictory. In some chronic infections, Tregs may play a beneficial role for the host. For instance, in case of infections mediated by lymphtrophic viruses (e.g., human immunodeficiency virus, HIV), Tregs limit hyperactivation of effector CD4^+^ T cells, thereby indirectly making them less susceptible to infection (154). Interestingly, it was reported that Tregs can inhibit the spreading of HIV from DCs to T cells by interfering with their immunological synapse (155), and in line with this Schulze Zur *et al.* observed a positive correlation between the relative frequency of Tregs, HIV load and disease progression in infected individuals (156). Nevertheless, as a side effect, there is an inefficient virus control, which subsequently results in chronic infection (146, 157). In addition, the persistent activation of Tregs can be harmful to the host, since it was shown that the sustained TGF-β production in macaques, chronically infected with simian immunodeficiency virus, leads to collagen deposition in lymphoid organs, likely limiting the space for immune reconstitution (158).

These opposing effects of Tregs could be explained by differentiating between acute and chronic infection (159). Early after infection the number of infected cells is still limited, thus the high Tregs:T effector ratio may be beneficial for the host. Instead, during a chronic infection, the number of infected cells is much higher, therefore Tregs may not be able to completely inhibit their proliferation, thus becoming detrimental for the host due to suppression of anti-pathogen responses.

It is well documented that the elderly population is more susceptible to different types of viral and bacterial infections, including influenza, pneumococcal disease, and herpes zoster. Both the prevalence and severity of these infections are notably increased among older adults compared to younger individuals, and they also suffer from more acute and long-term sequelae as a result (160).

Multiple studies have established correlations between age-dependent quantitative and qualitative changes in the Treg compartment and negative prognosis upon infection (161–164). Nevertheless, there is still a lack of a comprehensive mechanistic understanding of how these age-related changes in Treg function affect responses to infections. For instance, Lages *et al.* demonstrated that the presence of increased Treg numbers in elderly mice is responsible for the lack of an appropriate response against *Leishmania major*, which is likely due to the highly effective aged Treg-mediated suppression of IFN-γ production by Teff cells (66, 165). They also observed that chronic reactivation of this infection occurs more frequently in old mice compared to their younger counterparts as, upon healing, only 5% of the infected young mice exhibited clinical signs of lesion reactivation, whereas 75% of the infected old mice showed spontaneous reactivation (66). Of particular interest, the group demonstrated a direct role of Tregs in such reactivation, as *in vivo* depletion of Tregs in old mice attenuated disease severity and increased the production of IFN-γ by Teff cells at the infection site (66).

In addition to leishmaniasis, there are many other infectious diseases caused by pathogens that also produce persistent and latent infections. The reactivation of these infections causes an immense burden on health, especially in the immunosenescent population, where they can lead to recurrent diseases of increasing severity. A typical example is varicella zoster virus (VZV) infection, known also as chickenpox. VZV belongs to the α-herpesvirus family and usually causes infections during childhood. Despite a robust primary immune response, VZV is not eradicated and becomes persistent and latent. Virus reactivation, typically observed in adults, leads to shingles or herpes zoster and can also result in even more severe complications, including post-herpetic neuralgia (166). The mechanisms underlying virus reactivation are still to be clarified. However, a study in humans has shown that upon intradermal challenge with VZV, there is not only a local increase in VZV-specific CD4^+^ T cells, but also in Tregs. Although the authors did not assess the suppressive activity of these cells *in vivo*, they observed an inverse correlation between the augmented number of Tregs and reduced responses to the VZV challenge, indirectly suggesting that Tregs may be involved in VZV reactivation in older individuals by suppressing VZV-specific immune responses below a critical level (167).

Seasonal influenza viral infections represent another huge problem among the geriatric population both in terms of morbidity and mortality. Global estimates of seasonal influenza-associated mortality span from 300,000 to 650,000 deaths per year, with the highest at-risk group being people over the age of 75 (168). Notably, for influenza among other infectious diseases, substantial evidence has been provided regarding the correlation between aging, Tregs, and infection susceptibility. It was first reported that following influenza infection, aged mice display both a reduced and delayed immune response, evaluated by specific CD8^+^ T cell expansion and IFN-γ production (169). Subsequently, studies from Williams-Bey et al. revealed that following influenza infection, aged mice showed a significant expansion in their Treg pool, whereas young mice did not. Considering that old mice already start with a higher basal level of Tregs under homeostatic conditions compared to young mice, this means that upon influenza infection, their Treg numbers become much higher still. Thus, even though Tregs isolated from young and old mice do not show differences in terms of suppressive abilities, the age-dependent accrual of Tregs post-infection is sufficient to suppress IFN-γ production by anti-viral CD8^+^ Teff cells, thus hindering the immune response to influenza (80).

Recent studies by Morales-Nebreda *et al.* have reported that acute respiratory viral infections can lead to persistent pro-inflammatory and pro-fibrotic responses in the lungs of aged mice, and Tregs seem to play a major role in this complication (170). Several groups have highlighted a beneficial role played by Tregs in the context of lung infections. Aside from their role in maintaining immune homeostasis, Tregs can also accumulate in the lungs in response to viral injury to promote tissue repair, taking part in the resolution and healing phase following influenza infection (89, 171–175). One of the mechanisms by which they sustain tissue regeneration and repair is through the release of pro-resolutive mediators, including AREG (176). Unfortunately, in aged mice, this beneficial role of lung-resident Tregs is lost, since aging results in a cell-autonomous impairment of repair-type Treg function during influenza pneumonia (170). Specifically, researchers attributed the loss of youthful pro-resolutive functions, at least in part, to age-related modifications in the Treg methylome, which affect their reparative transcriptional regulatory network and consequently result in a prolonged recovery phase post-influenza infection (170).

Interestingly, this age-dependent Treg dysregulation in the healing program may also contribute to the prolonged recovery and worse clinical outcomes, including severe disease and death, observed in aged hosts upon SARS-CoV-2 infection compared to younger individuals (177). In addition to the discovery that COVID-19 patients with more severe disease had more circulating Tregs compared to milder cases, it was found that these Tregs were also transcriptionally distinct, including an increased expression of FOXP3 and Notch4, a molecule associated with increased lung inflammation and decreased tissue repair (178–180). Furthermore, severe disease-associated Tregs displayed overexpression of various suppressive effectors and proinflammatory molecules indicative of strong activation, with the exception of the pro-resolutive mediator AREG (179, 180). Overall, Tregs from COVID-19 patients with severe disease tend towards a super-suppressive phenotype and lack promotion of tissue repair (179).

The role of Tregs in COVID-19 immunopathogenesis is so far incompletely understood, and the literature is often conflicting and controversial. For instance, several studies have reported an increased frequency of Tregs in severe COVID-19 cases (178, 179, 181–184). In contrast, others revealed a reduced proportion of SARS-CoV-2-reactive Tregs in hospitalized and severel diseased patients (185–188), and based on these observations, some authors have proposed the use of Tregs as a therapeutic tool for COVID-19 patients with acute respiratory distress syndrome (ARDS) (189). Notably, proportions of Tregs in COVID-19 patients are not significantly different to proportions in patients infected with respiratory syncytial virus or influenza, and the underlying reason why Treg function results in different symptomatic outcomes in the elderly for these diseases remains to be elucidated (178). It is possible that the role of Tregs evolves throughout COVID-19 disease progression: an increase in Tregs early in infection may hinder a potent antiviral immune response, whereas fewer Tregs or dysfunctional Tregs later in infection may result in an overactive inflammatory cytokine storm (190, 191). Tahmasebi et al. examined the effect of the anti-inflammatory therapeutic Nanocurcumin on Treg populations in severe COVID-19 cases associated with reduced Treg numbers. They found an increase in Tregs and their mediated factors, such as TGF-β, IL-10 and IL-35, which resulted in a significant reduction in associated clinical symptoms, most likely by dampening the harmful cytokine storm (192). Collectively, the role of Tregs in the immune response against SARS-CoV-2 is not clear yet and remains to be clarified with more detailed studies.

These data highlight the controversial role of Tregs in infections. On one hand, the increased number of Tregs hinders immune responses against invading pathogens, thus playing a detrimental role in acute and chronic infections. On the other hand, they might be useful in the resolution phase post-infection by promoting tissue repair, but they display a dysfunctional phenotype, thus contributing to the prolonged recovery phase in older patients.

## 6 Role of Tregs in poor vaccine response in the elderly

Prophylactic medicine is an effective strategy to protect the health of older individuals, and vaccination is the most effective and widespread approach for conferring protection against (re)infections, thus reducing pathogen-associated disease progression (193). At least four vaccines against infectious agents are currently recommended to the elderly, including vaccines against influenza, pneumococcal disease, herpes zoster, as well as regular booster shots against tetanus, diphtheria, and pertussis (194). However, primary vaccine responses are often poor in older people, and fail to induce long-term protective immunity, thereby placing these individuals at further risk for subsequent disease (195).

Aging is associated with a decline in the production of high-affinity neutralizing antibodies after vaccination (196, 197). These antibodies, as well as immunological memory, are produced as a result of vaccine-induced GC reactions and constitute the basis of successful immunization. GCs are transient specialized microstructures, typically originating within lymphoid follicles, where B cells undergo affinity maturation and genetic diversification of their B cell receptor (BCR) repertoire before differentiating into long-lived, antibody-secreting plasma cells and memory B cells (198). The first few days after the initiation of a T cell-dependent B cell response, activated extrafollicular B cells undergo class-switch recombination (199) and then return to the follicle, where they proliferate and go through somatic hypermutation, giving rise to the dark zone (DZ) of the GC (200, 201). The B cell progeny then migrate to the light zone (LZ) of the GC, where they interact with follicular dendritic cells and primed antigen-specific T_FH_ cells to undergo a positive selection process (202–204). Ultimately, only high-affinity B cells will be positively selected and will differentiate into antibody-secreting plasma blasts and memory B cells (198, 205–207).

**Figure 2:**
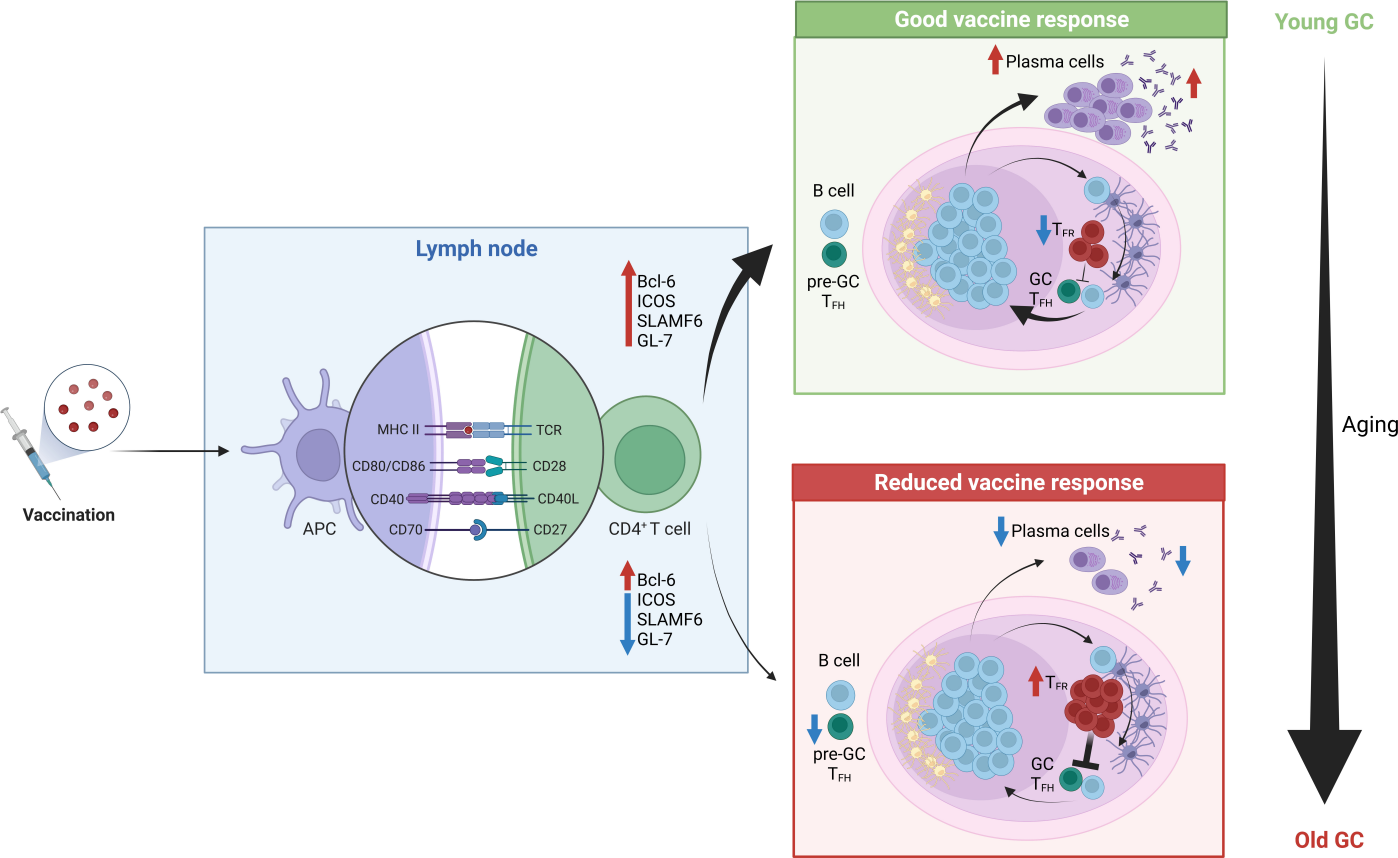
Reduced vaccine response in the elderly. Upon antigenic stimulation, antigen presenting cells capture, process and present the antigen to naïve CD4^+^ T cells in the draining lymph node. Upon the formation of an immunological synapse between these two cell types, in young individuals naïve CD4^+^ T cells get efficiently activated and acquire a T_FH_-like phenotype by up-regulating Bcl-6, ICOS, SLAMFF6 and GL-7. Consequently, they migrate to the GC, where they will sustain a T cell-depedent B-cell response. Finally, mature plasma cells will be generated, and they will produce high-affinity antibodies against the antigen, thereby providing a good vaccine response. In contrast, in older individuals typically a scares response to vaccines is observed. Among the possible causes, it was found that activated CD4^+^ T cells failed to up-regulate some T_FH_ cell markers, which are crucial to promote migration towards the GC, thus a reduced amount of T cells reach this location. Moreover, upon antigenic stimulation, a higher frequency of T_FR_ cells can be found in the GC, which inevitably results in the suppression of this T cell-dependent B cell responses.

During aging, the GC and T_FH_ cell response declines in quality, resulting in impaired humoral immunity (208, 209) (**Figure 2**). In human vaccination studies fewer circulating T_FH_-like cells, that are considered biomarkers of the GC reaction, are observed in older individuals (17, 18). This phenomenon has sparked considerable interest in the discovery of the mechanisms underlying this poor response. For instance, animal studies have revealed that T_FH_ cell differentiation is impaired by aging with T_FH_ cell formation possible, but full differentiation into GC T_FH_ cells compromised (165, 210). Lefebvre *et al.* observed that activated influenza-specific CD4^+^ T cells were able to upregulate the transcription factor B-cell lymphoma 6 (Bcl6), thus becoming T_FH_-committed cells in the aged host. However, these cells failed to upregulate the expression of some T_FH_-specific molecules like ICOS, SLAMF6 and GL-7, which is required for migration into the GCs where T cell-dependent antibody responses take place. Instead, they are characterized by an enhanced production of IL-10 and IFN-γ (165). Altogether, these data indicate that the interaction between T_FH_ cells and cognate B cells might be impaired in the aged host. Similar findings were also found in recent studies combining mice and humans, which identified an impaired differentiation of T_FH_ cells upon vaccination in the elderly, due to suboptimal T cell priming by conventional type 2 DCs, consequently resulting in poor GC B cell expansion (211).

In addition to T_FH_ cells, T follicular regulatory (T_FR_) cells are also present in the GC. T_FR_ cells are a subset of Tregs with specialized roles in regulating T_FH_-driven GC responses, preventing the induction of autoreactive and foreign antigen-specific antibodies (212). T_FR_ cells can originate from tTregs (213) or from Tconv *via* the induction of Foxp3 expression prior to T_FR_ differentiation (214). Interestingly, it has been demonstrated that following antigen immunization, aged mice develop a higher percentage of T_FH_ and T_FR_ cells, with proportionally more T_FR_ cells, compared to young mice (215). Sage *et al.* suggested that this preferential accumulation of T_FR_ over T_FH_ cells is a consequence of the high basal level of Tregs found in elderly individuals when compared to their younger counterparts. However, increased T_FR_ cell numbers are not observed in all studies of aged mice after vaccination, suggesting that it may be possible to change vaccine type in order to avoid excess T_FR_ cell formation (216). Lefebvre et al. have observed that at the peak of the GC response, 14 days post-infection (dpi) following a sublethal dose of influenza A virus, the number of nucleoprotein-specific Treg and T_FR_ cells was significantly higher in aged compared to young mice. This suggests they may play a role in the poor humoral response observed in aged mice after influenza infection (165). In addition, the group adoptively transferred young polyclonal CD4^+^ T cells into young and aged mice one day prior to influenza infection, and the analysis performed 14 dpi revealed an enhanced differentiation of the donor cells toward a T_FR_ cell phenotype in aged mice. Interestingly, this was accompanied by a higher level of active TGF-β1 (a well-known inducer of Foxp3 expression) in the spleens of aged compared to young mice, further suggesting that the aged environment may also drive increased Treg generation in aged hosts upon antigen stimulation (165). Despite young and aged T_FR_ cells having similar suppressive capacity on a per-cell basis *in vitro* and *in vivo*, this skewing of the T_FH_/T_FR_ ratio towards suppressive T_FR_ cells was postulated to contribute to the impaired GC response in aging (215). However, halving the number of T_FR_ cells in the GC through deletion of CXCR5 (217) did not enhance the mangnitude of the GC response in aged *Cxcr5* ^fl/fl^ *Foxp3* ^Cre^ mice, compared with their aged littermate controls (217), indicating that the accumulation of T_FR_ cells is not the primary cause of diminished GC reactions in aging.

In light of the ongoing COVID-19 pandemic, during which older demographics have been disproportionately afflicted in terms of disease severity, improving vaccines for the elderly has become a top priority (218–221). Of the several vaccine strategies against COVID-19 that exist, many show similar trends when investigating age-related differences of efficacy. While the mRNA vaccines including BNT162b2 (BioNTech/Pfizer) and Moderna 1273, as well as the adenoviral vectored ChAdOx1 nCoV19 vaccine (AstraZeneca), have been shown to elicit robust responses in young recipients, this efficacy is profoundly reduced in older patients, for whom booster immunisations are essential for protection against COVID-19 (216, 222–224). While the literature focusing on COVID-19 vaccination-induced Tregs in the elderly is scarce, these trends mirror those seen with other vaccine regimes, including influenza and herpes zoster (224, 225). For instance, after influenza immunization in humans, non-responders present higher frequencies of total and naïve Tregs than their vaccine-responding counterparts (226). In addition, Uraki et al. demonstrated in mice that a transient reduction in Tregs during a COVID-19 vaccine-specific GC response led to an increase in both DCs and T_FH_ cells, further corroborating the notion of Tregs dampening the immune response, as is seen in older individuals (227). Fully understanding how Tregs contribute to the immune response upon immunization may inform about how to design vaccines in the future to elicit better immune responses in the more vulnerable elderly.

Overall, there are both CD4^+^ T cell-intrinsic and extrinsic factors that contribute to the regulatory environment of aged mice and humans, leading to the suppression of T_FH_ cell function and subsequent weakening of the GC response in aged individuals. Since it is unlikely that an age-related increase in the T_FR_/T_FH_ ratio alone can affect the GC response (217, 228), it remains to be fully understood how aging of the GC contribute to reduced vaccine responses in aged hosts.

## 7 Treg modulation to improve vaccine effectiveness in the elderly

The rapid expansion of the aging population worldwide, together with the reduced response to vaccines observed among the elderly, have pushed for the research of new strategies to optimally elicit long-lasting immune responses in this age group. Although the scientific research in this field is still limited, different strategies are currently in place to improve vaccine effectiveness in old people, primarily focusing on the use of higher doses of antigens, adjuvants, alternative administration routes, immunomodulatory drugs and senolytics, which are small molecule drugs intended to selectively induce the death of senescent cells (194).

In recent years, there has been increasing interest regarding the modulation of Tregs to improve the immunogenicity and effectiveness of vaccines. This is primarily due to growing evidence suggesting that the enhanced age-dependent Treg expansion and activation during immunization may be partially responsible for the reduced effectiveness of certain vaccines. For instance, the depletion of these suppressive cells *via* injection of anti-CD25 antibodies has been proven to be effective in restoring the vaccine-induced immune responses in mice (229). Accordingly, higher vaccine dosage was required to confer complete protection against influenza virus infection in aged mice, and depletion of Tregs using anti-CD25 antibody treatment prior to influenza infection protected against lethal viral challenge in aged mice, instead leading to significantly increased levels of specific antibodies (24). Consistent with this, Yang *et al.*, using an influenza virus infection mouse model with the adoptive transfer of TCR-transgenic CD4^+^ T cells, demonstrated that primary vaccination with unadjuvanted peptides induced a significant portion of antigen-specific Tregs, which were further expanded upon secondary vaccination (230). The suppressive role of these cells was also demonstrated *in vivo*, as specific depletion of vaccine-induced Tregs favored viral clearance. Interestingly, the group discovered that immunization with CpG-adjuvanted peptides by subcutaneous prime-intranasal-boost strategy stimulated robust T cell immunity and restricted the recruitment and accumulation of Tregs in the lungs. These results indicate that with the use of certain adjuvants it is possible to modulate and reverse the detrimental effects of Tregs during immunization.

Given the common deleterious effects of these cells in the vaccine-induced immune response, several Treg-modulating approaches have been developed and are currently in use to generate vaccines tailored for old people. These approaches are based on Treg transient depletion or inhibition, using molecular adjuvants during the induction phase of the immune response (231). Currently, monoclonal antibodies (mAbs) are the most widely used Treg modulators for vaccine improvement. Besides anti-CD25, other mAbs have been approved for clinical use, targeting other Treg extracellular markers (e.g. CTLA-4, PD1, ICOS, LAG3, TIM3, GITR) but also Treg cytokines (e.g. TGF-β, IL-10, and IL-35) (231). However, different concerns about the use of mAbs as vaccine adjuvants remain. For instance, they have a relatively long lifespan, thus the effect on Tregs can be long-lasting, in turn potentially facilitating the development of autoimmune and other associated immunotoxic reactions. Moreover, there is the possibility of cross-reactivity causing off-target effects, the inability to reach intracellular targets, and the technological complexities and high costs associated with their pharmacological production (232). Nevertheless, many other Treg-modulating alternatives to mAbs are currently being evaluated as molecular adjuvants, including synthetic peptides. For instance, P60 is a small peptide that can enter the cells, bind to Foxp3, and subsequently blocks its nuclear translocation, thus reducing its ability to suppress the transcription factors NF-kB and NFAT. In this way, P60 elicits Teff cell stimulation and inhibition of murine- or human-derived Tregs (233, 234). Alternatively, antisense oligonucleotides (ASOs) have also been proposed as a novel strategy to improve the effectiveness of vaccines (235). A recent study used a model of intradermal vaccination with OVA to demonstrate that ASO-mediated knockdown of IL-10 is a safe and potent way to improve vaccines. Specifically, the use of the IL-10-specific ASOs resulted in a 100-fold increase in anti-OVA antibody titers, associated with an enhanced T cell-mediated specific immune response (236). Furthermore, TGF-β2-specific ASOs have been formulated for use in various microbial vaccines, which have significantly enhanced the specific antibody response to vaccines (237).

The advantages of these biomolecules include the availability of more formulation and delivery options, lower molecular complexity, and shorter pharmacokinetic half-life, thus a reduced risk of adverse reactions is expected. These major advantages are very attractive and provide the basis for developing combinatorial therapeutics to ameliorate immunization and gain more efficient control of bacterial and viral infections in the elderly.

## 8 Conclusion

Aging is a very complex physiological phenomenon characterized by a plethora of biological changes including those affecting the immune system, such as thymic involution, immunosenescence and inflammaging. These are believed to be the underlying causes behind the increased susceptibility of the old population to cancers, infections and autoimmune diseases, as well as the reduced response to vaccinations.

Tregs plays a fundamental role as regulators of different immune responses, keeping autoreactive and/or exaggerated immune responses in balance. Thus, the age-related changes affecting this cell compartment are of particular interest. Despite a reduced thymopoiesis and peripheral differentiation of Tregs during aging, several data show accumulation of Tregs in SLOs both in mice and humans. This has been attributed to an enhanced survival of old Tregs compared to their young counterpart, due to the selective loss of expression of the pro-apoptotic protein Bim. Beside this quantitative changes, many studies have been performed to evaluate Tregs’ immunosuppressive functions during aging. However, results are often inconsistent and opposing. This is likely due to the lack of a standardized method to evaluate Treg functionality, therefore results are significantly affected by several confounding variables.

Current data on age-related quantitative and qualitative changes of Tregs do not completely explain the simultaneous increased risk of autoimmunity, cancer, and infections in the elderly. The number of Tregs plays a decisive role in the whole regulation of the immune system as too many Tregs can trigger excessive immune suppression, while too few can lead to autoimmune responses. In this regard, the age-dependent Tregs accrual might explain why old people are more prone to develop infections and neoplastic malignancies. Whereas the higher susceptibility to autoimmune diseases would agree with Treg dysfuction, i.e. enhanced Treg functionality. Overall, knowing that both Treg numbers and functions are crucial for a proper regulation of the immune system, it is possible that both are affected during aging but in a different way in each individual (inter-individual variability in the aging process). Moreover, we should always take into account that aging is a complex, system-wide, multi-factorial phenomenon, thus trying to explain these contradictory results by focusing only on a cell type (Tregs in this case) might be a too reductionist approach. Immune aging is more than a functional degeneration of a single cell type, wherefore we should consider also the impact of other immune cells, genetics and environmental factors to have a more complete view of the aging immunobiography of each individual.

Despite many advancements in the understanding of the role of Tregs during aging, this review highlights a range of open questions for the future, including *i)* Which specific molecular mechanisms govern Treg numbers with age? *ii)* How can we properly assess Treg function in the context of aging? *iii)* What happens to tissue-resident Tregs during aging? *iv)* Which other Treg-specific molecular targets will help us to ameliorate vaccine responses and reduce predisposition to multimorbidity in the elderly?

Further studies will illuminate nuances in Treg heterogeneity, so that similar populations can be compared between young and aged subjects when assessing their activity. Finally, animal models will be invaluable assets to unravel age-driven dysregulation in tissue-resident Tregs. Ultimately, this research may pave the way to developing novel therapeutic strategies tailored to manipulate local Treg accumulation and function to selectively enhance (e.g. antitumor immunity, infections, vaccination) or inhibit (e.g. autoimmunity) Teff cell responses to promote healthy aging.

## Author contributions

All authors contributed to the design and writing of this review. All authors contributed to the article and approved the submitted version.

## Funding

This project has received funding from the European Union’s Horizon 2020 research and innovation programme under the Marie Skłodowska-Curie grant agreement No 955321 and from the Deutsche Forschungsgemeinschaft (DFG, German Research Foundation) under Germany’s Excellence Strategy - EXC 2155 - project number 390874280.

## Acknowledgments

We thank Frauke Sawadda for proof-reading. Cartoons in **Figures 1**, **2** were created with Biorender.

## Conflict of interest

The authors declare that the work was conducted in the absence of any commercial or financial relationships that could be construed as a potential conflict of interest.

## Publisher’s note

All claims expressed in this article are solely those of the authors and do not necessarily represent those of their affiliated organizations, or those of the publisher, the editors and the reviewers. Any product that may be evaluated in this article, or claim that may be made by its manufacturer, is not guaranteed or endorsed by the publisher.
